# Ex Vivo Evaluation of Buserelin Permeation Across Porcine Endometrial Tissue: A Pilot Study

**DOI:** 10.3390/ani16193108

**Published:** 2026-10-03

**Authors:** Preechaphon Taechamaeteekul, Romchat Chutoprapat, Robert V. Knox, Hongyao Lin, Miquel Collell, Padet Tummaruk

**Affiliations:** 1 Department of Obstetrics, Gynaecology and Reproduction, Faculty of Veterinary Science, Chulalongkorn University, Bangkok 10330, Thailand; taechamaeteekul.p@gmail.com; 2 Department of Pharmaceutics and Industrial Pharmacy, Faculty of Pharmaceutical Sciences, Chulalongkorn University, Bangkok 10300, Thailand; romchat.c@pharm.chula.ac.th; 3 Department of Animal Sciences, University of Illinois, Champaign-Urbana, IL 61801, USA; rknox@illinois.edu; 4 Merck Animal Health, 2 Giralda Farms, Madison, NJ 07940, USA; hongyao.lin@msd.com (H.L.); miquel.collell@merck.com (M.C.); 5 Centre of Excellence in Swine Reproduction, Chulalongkorn University, Bangkok 10330, Thailand; 6 The Academy of Science, The Royal Society of Thailand, Bangkok 10210, Thailand

**Keywords:** artificial insemination, Franz diffusion, permeability, pig, uterine tissue

## Abstract

Buserelin, a gonadotropin-releasing hormone (GnRH) analog, is commonly used in swine to induce ovulation and facilitate fixed-time artificial insemination (FTAI). Supplementation of diluted semen with buserelin has been developed as a less invasive approach to synchronize ovulation and potentially enhance litter size in gilts. This study evaluated the diffusion of buserelin across porcine endometrial tissue using an ex vivo Franz diffusion cell system. The primary objective was to determine whether buserelin, either alone or in combination with extended boar semen, could penetrate sow uterine tissue. Endometrial tissues were collected post-mortem from six sows confirmed to be in the follicular phase by macroscopic examination of the reproductive tract. Each uterine sample was divided into two portions and assigned to either a buserelin group or a buserelin plus diluted semen group. Boar semen used for the diluted semen group was prepared using a commercial extender, and the pH of all test samples was measured prior to analysis. Diffusion studies were performed using vertical Franz diffusion cells maintained at 37 °C, with phosphate-buffered saline (pH 7.4) serving as the receptor medium. To standardize the total drug dose applied (rather than the concentration or application volume), tissue samples were treated either with 4 µg of buserelin acetate in 1 mL of solution or with 4 µg of buserelin acetate in 1 mL of solution mixed with 1 mL of diluted semen. Receptor fluid was collected over an 8-h period for quantification of permeated and absorbed buserelin using high-performance liquid chromatography (HPLC). The overall percentage of buserelin permeation through porcine endometrial tissue was similar between the buserelin group (61.3 ± 7.8%; range 54.0–73.7%) and the buserelin plus diluted semen group (62.2 ± 4.6%; range 48.6–68.9%), with no significant difference (*p* = 0.840). During the first 0.75 h (0–45 min), the cumulative permeation did not differ between treatments (10.2 ± 2.4% vs. 11.8 ± 2.4%, *p* = 1.000). However, at 1.5 h, the permeation rate was lower in the buserelin group than in the buserelin plus diluted semen group (18.0 ± 2.4% vs. 29.2 ± 2.4%, *p* = 0.032). From this point onward, the buserelin plus diluted semen group maintained higher permeation until approximately 6 h, after which permeation values became comparable (*p* = 0.174). The steady-state fluxes (*J*_ss_) were 0.21 and 0.28 µg/cm^2^·h, with corresponding apparent permeability coefficients (*P*_app_) of 0.052 and 0.069 cm/h, respectively. In conclusion, this ex vivo pilot study showed that more than 61% of the administered buserelin permeated porcine endometrial tissue within 8 h, irrespective of semen supplementation. The addition of diluted semen enhanced permeation during the early phase but did not affect the overall cumulative diffusion.

## Simple Summary

Buserelin is a synthetic hormone used in pig farming to help control the timing of egg release and plan insemination. It is usually given by injection, but adding it directly to semen could provide a simpler and less invasive alternative. For this approach to work, the hormone must first pass through the uterine lining. We investigated whether buserelin could pass through uterine tissue collected from six sows, either alone or mixed with diluted boar semen. The tissue was placed in laboratory chambers, and the passage of buserelin was measured over 8 h. More than 61% of the applied hormone passed through the tissue under both conditions. These findings provide preliminary evidence that buserelin can cross sow uterine tissue in the presence or absence of semen. Further studies in live animals are needed to determine whether enough hormone reaches the bloodstream to control egg release effectively.

## 1. Introduction

Gonadotropin-releasing hormone (GnRH) analogs are increasingly applied in swine production to induce ovulation and enable fixed-time artificial insemination (FTAI) [1,2,3]. These synthetic hormones are traditionally administered via the intramuscular route [1,4]. Alternatively, a non-invasive intravaginal delivery method has been developed and is widely used in the United States [5]. More recently, buserelin, a GnRH analog, has been incorporated directly into extended fresh semen for artificial insemination (AI) to stimulate ovulation and improve litter size in both gilts [6] and sows [7,8].

Studies in pigs and cows have demonstrated that buserelin is sufficiently stable to maintain therapeutic levels following intramuscular administration [9,10]. No adverse effects have been reported, confirming its in vivo stability throughout the pharmacokinetic period [9,10]. A recent study showed that the time to reach maximum plasma concentration of buserelin (Receptal) was 0.30 ± 0.10 h, and the elimination half-life was 0.51 ± 0.03 h [10]. Recently, the incorporation of buserelin into boar semen has gained attention for its potential to enhance farm productivity by increasing litter size in gilts [8]. However, to our knowledge, no study has evaluated the mechanism by which buserelin penetrates the sow endometrium. Therefore, understanding the ability of buserelin to permeate endometrial tissue is crucial for optimizing its delivery method and for verifying the hypothesis that the addition of buserelin to boar semen can improve litter size in gilts [6] and sows [7].

The Franz diffusion cell technique is widely recognized as a reliable method for evaluating drug permeability across biological barriers, including skin and mucosal tissues [11,12]. This ex vivo model provides valuable insights into drug–membrane interactions and the potential for transmembrane delivery, particularly for peptide-based hormones such as GnRH agonists [3,13]. When applying this method to porcine endometrial tissue instead of synthetic membranes or skin, it is important to consider that structural and functional differences among tissue types can influence diffusion kinetics [14,15]. The skin, composed of the epidermis, dermis, and subcutaneous layers, serves primarily as a protective barrier, whereas the endometrium, the inner lining of the uterus, consists mainly of epithelial and stromal cells [16,17,18]. Recognizing these anatomical distinctions is essential for developing tissue-specific experimental protocols [19]. Nevertheless, previous studies have demonstrated that using reproductive tissues such as the vagina and endometrium in Franz diffusion cell systems offers distinct advantages and represents a promising model for assessing drug permeability [20,21,22].

A previous study demonstrated buserelin permeation through vaginal tissue using the Franz diffusion cell method, reporting a maximum flux of 0.6 × 10^−2^ µg/cm^2^·h and an apparent permeability coefficient of 16.2 × 10^−5^ cm/h. These results highlight the ability of buserelin to traverse vaginal tissue in sows and support its use as a model for evaluating local drug delivery in swine [22]. However, to date, no study has examined the permeation of buserelin across the sow endometrium. In practical swine reproduction, buserelin is typically administered at the onset of estrus or when follicle diameter exceeds 6.5 mm to induce ovulation, which occurs at the end of the follicular phase [1,23,24,25]. This underscores that the optimal timing for buserelin administration in gilts and sows corresponds to the late follicular phase. To mimic these physiological conditions, endometrial tissue used in permeability testing should be collected during the follicular phase, as structural and functional changes in the endometrium during this period may significantly influence drug transport [22,23]. Therefore, the present study aimed to determine whether buserelin, either alone or mixed with extended boar semen, could cross porcine endometrial tissue collected during the follicular phase, using an ex vivo Franz diffusion cell system as a model of transport across the uterine lining.

## 2. Materials and Methods

### 2.1. Animals and Experimental Design

This study was conducted in accordance with the ethical principles and guidelines for the use of animals in scientific research established by the National Research Council of Thailand. All procedures were approved by the Institutional Animal Care and Use Committee (IACUC) of Chulalongkorn University (Approval No. 2331095), in compliance with institutional policies and regulations governing the care and use of experimental animals. This study evaluated the diffusion of buserelin acetate (Receptal^®^, Merck Animal Health, Rahway, NJ, USA) across porcine endometrial tissue using an ex vivo Franz diffusion cell system. Endometrial tissues were collected post-mortem from six sows obtained from a slaughterhouse in Thailand. Uterine tissues were harvested immediately after slaughter, transported in a chilled Styrofoam container, and delivered to the necropsy room within 12 h. The tissues were subsequently dissected and stored at −20 °C until used in the Franz diffusion assay. All sows (parity 2–4; *n* = 6) were culled for management-related reasons and showed no evidence of reproductive disorders. Reproductive tracts were macroscopically examined to confirm the follicular phase based on endometrial edema, cervical rigidity, and the presence of large ovarian follicles (≥6.0 mm), indicative of estrus activity [26]. Only tissues confirmed to be in the follicular phase were included, as artificial insemination, and thus buserelin application, occurs during this period [1].

### 2.2. Sample Preparation

Each uterine horn was sampled near the main blood vessels and sectioned into two proximal portions within 30 cm of the uterine body junction. The resulting 12 samples were randomly assigned to either the buserelin group (*n* = 6) or the buserelin plus diluted semen group (*n* = 6). The endometrial layer was carefully dissected from the underlying myometrium and trimmed into discs of 2 cm diameter, to an approximate thickness of 1.0 mm, in order to fit the Franz diffusion cell. Dissection was performed by a single operator throughout so that samples of comparable thickness were obtained. Samples were immediately immersed in cold phosphate-buffered saline (PBS; pH 7.4), wrapped in aluminum foil, and stored at −20 °C until use. Prior to the experiment, tissues were thawed at room temperature (25 °C). This exploratory ex vivo pilot study used storage at −20 °C to preserve tissue architecture before diffusion testing and to follow previously published protocols [27,28].

To ensure a consistent total drug dose across treatments, each tissue sample received 4 µg of buserelin acetate. Samples were allocated to two groups: (i) the Buserelin group, in which 4 µg of buserelin was applied in 1 mL of solution (Receptal^®^, Merck Animal Health, Rahway, NJ, USA), and (ii) Buserelin + diluted semen group, in which the same 4 µg dose was delivered in a 1:1 mixture of buserelin solution (1 mL) and diluted semen (1 mL). For the preparation of the diluted semen, boar ejaculates were collected from an artificial insemination (AI) center at a commercial pig farm and transported to the laboratory within 3 h of collection. The semen was diluted with a commercial extender (MR-A^®^ 3; KUBUS LAB S.A., Madrid, Spain) to achieve a final sperm concentration of 33.3 × 10^6^ sperm/mL and stored at 16 °C until analysis, which was performed within 2 days. The pH of all test samples was measured using a pH meter (FiveEasy Plus, InLab Viscous Pro-ISM; Mettler Toledo Thailand Ltd., Bangkok, Thailand).

### 2.3. Franz Cell Diffusion

**Figure 1:**
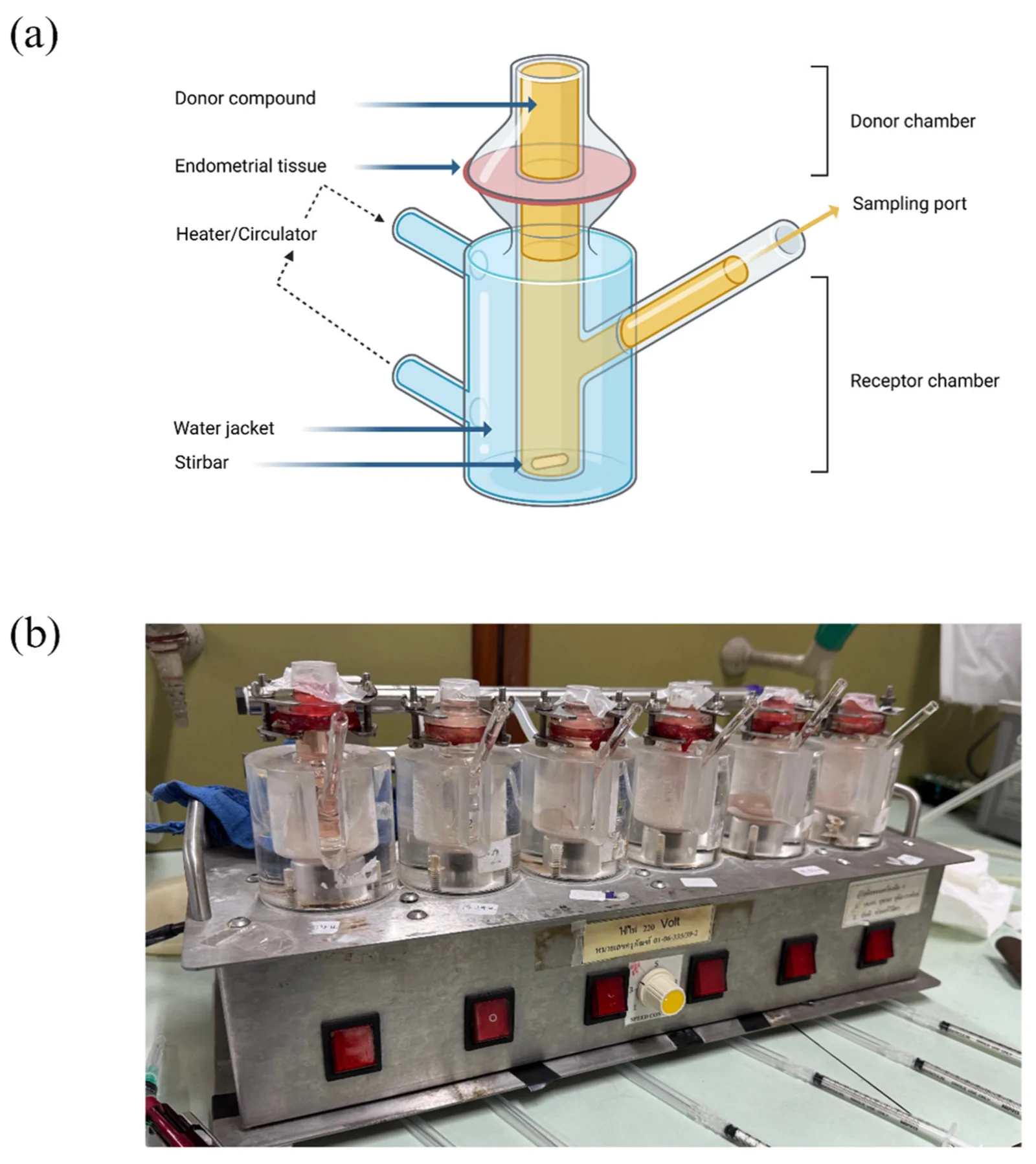
(**a**) Schematic illustration of the vertical Franz diffusion cell setup. (**b**) Picture of the Franz diffusion cell used in the experiment.

The permeation study of buserelin was conducted using vertical Franz diffusion cells (Figure 1). Endometrial tissues were mounted between the donor and receptor chambers, with the endometrial side facing the donor compartment containing the test compound. The tissues were carefully positioned on the glass surface of the Franz cell to ensure firm contact and to avoid air bubbles that could interfere with diffusion. The effective diffusion area of each cell was 0.79 cm^2^. An O-ring clamp was applied to secure the assembly and prevent leakage between chambers. The receptor chamber was filled with PBS and maintained at 37 °C to simulate physiological temperature. Prior to the experiment, the tissues were pre-equilibrated in the receptor buffer for 30 min to ensure stabilization. To standardize the total drug dose (rather than concentration or application volume), each tissue received 4 µg of buserelin acetate applied in either 1 mL of buserelin solution alone or 1 mL of buserelin solution mixed with 1 mL of diluted semen, resulting in initial concentrations of 4 µg/mL and 2 µg/mL, respectively. This difference in donor volume was intentional, reflecting the practical on-farm condition in which buserelin is added directly to extended semen, and comparisons between groups should therefore be interpreted in terms of total dose rather than concentration equivalence. Continuous magnetic stirring of the receptor chamber was maintained throughout the experiment to ensure homogeneity and facilitate accurate diffusion. Samples of the receptor fluid (0.7 mL) were withdrawn at 45 min, 1.5 h, 2 h, 3 h, 4 h, 5 h, 6 h, 7 h, and 8 h after initiation of the experiment. After each collection, an equal volume (0.7 mL) of fresh PBS was added to maintain a constant receptor volume. At the end of the 8-h diffusion period, each tissue sample was cut into small pieces along the tested surface and homogenized in 3 mL of 70% methanol (*v*/*v*) to extract the absorbed buserelin. The homogenate was vortexed for 1 min, sonicated for 5 min, and centrifuged at 10,000× *g* for 10 min at 4 °C. The resulting supernatant was collected for subsequent analysis. Residual fluid from the donor chamber was also collected for analysis. All collected samples, including receptor medium withdrawn at each sampling time, methanol extracts, and donor solutions, were filtered through a non-sterile disposable syringe filter with a nylon membrane (0.2 µm, Ø 18 mm; Whatman Uniflo^®^, GE Healthcare Life Sciences, Chicago, IL, USA) into 2-mL amber glass vials for further analysis.

### 2.4. Measurement of Buserelin Concentration

**Figure 2:**
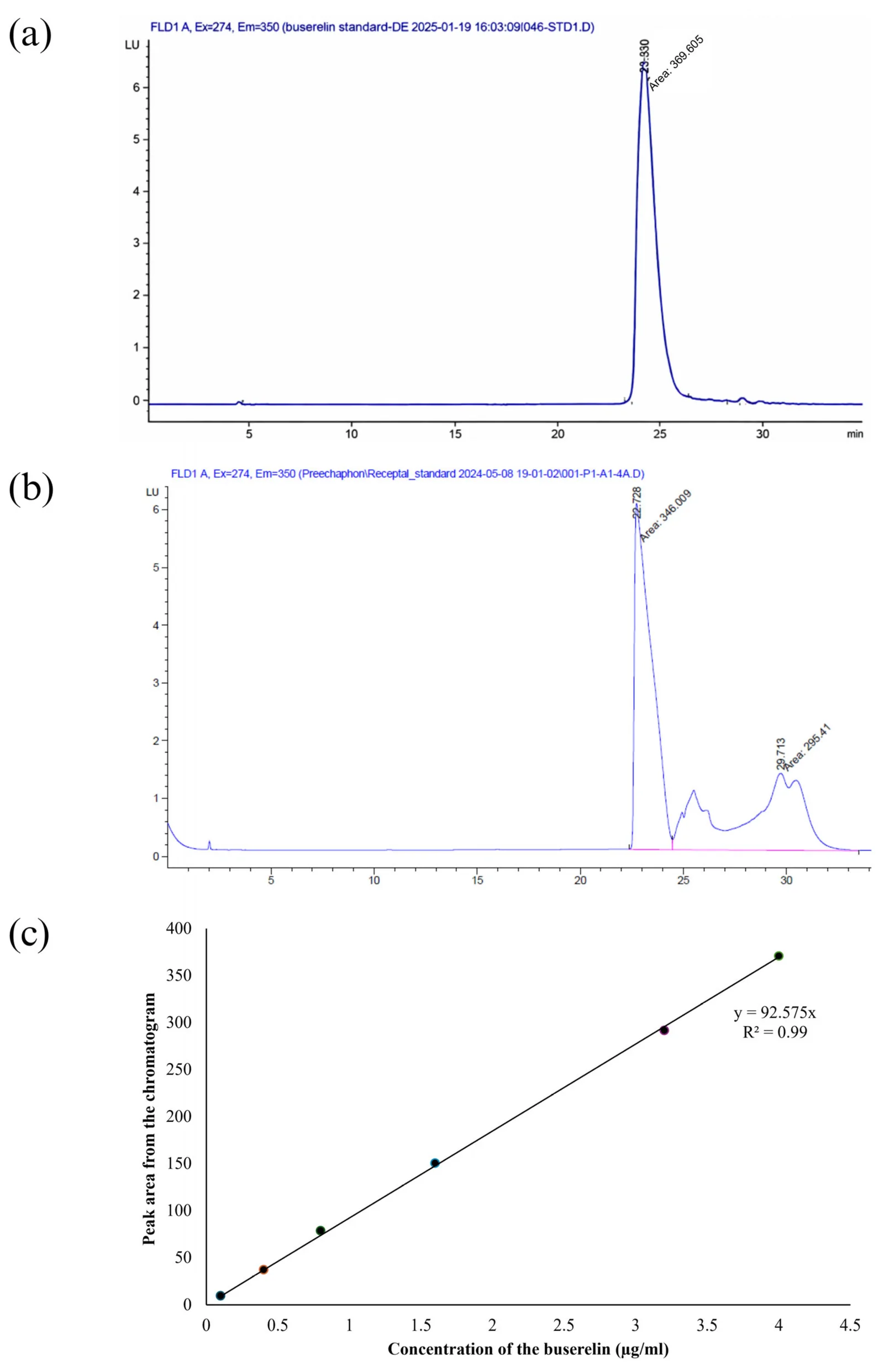
High-performance liquid chromatography (HPLC) chromatogram of standard buserelin showing an average retention time of 23.8 min (**a**) and Receptal^®^ with an average retention time of 22.7 min (**b**). (**c**) Linearity of the calibration curve for standard buserelin across six concentrations (0.1, 0.4, 0.8, 1.6, 3.2, and 4.0 µg/mL), demonstrating excellent analytical precision (*R*^2^ = 0.99).

The concentration of buserelin was quantified using high-performance liquid chromatography (HPLC). Analyses were conducted on an Agilent HPLC system equipped with a fluorescence detector and a Nucleosil C18 column (15 cm × 4.6 mm; Sigma-Aldrich, St. Louis, MO, USA). The method was adapted from Suszka-Świtek et al. [29]. Two mobile phases were prepared, Buffer A (acetonitrile:water, 5:95 *v*/*v*) and Buffer B (acetonitrile:water, 35:65 *v*/*v*), each containing 0.05% trifluoroacetic acid (HPLC grade, Fisher Chemical, Leicestershire, UK). All solutions, including mobile phases, were filtered through a 0.20 µm cellulose acetate membrane (Whatman™, Maidstone, UK) and ultrasonically degassed. Milli-Q purified water (18.2 MΩ·cm; Millipore Milli-Q, Darmstadt, Germany) was used throughout the analyses. Chromatographic separation was performed using the following gradient program: 0–10 min, 100% Buffer A; 10–20 min, Buffer A reduced to 83% and Buffer B increased to 17%; 20–27 min, Buffer A reduced to 0% and Buffer B increased to 100%; 27–30 min, Buffer A returned to 100%; and 30–35 min, conditions held at 100% Buffer A. The flow rate was 0.8 mL/min, with a 10 µL injection volume, and the column temperature was maintained at 28 °C. Buserelin was detected by fluorescence at excitation and emission wavelengths of 274 and 350 nm, respectively. The mean retention times were 23.8 min for the buserelin standard (Figure 2a) and 22.7 min for Receptal^®^ (Figure 2b). A buserelin reference standard was injected in each analytical sequence, and the analyte peak was assigned by comparing its retention time with that of the reference standard analyzed in the same sequence. Between-sequence variation in the retention time of the reference standard was monitored.

For calibration, HPLC-grade buserelin (Sigma-Aldrich Fine Chemicals Biosciences, St. Louis, MO, USA) was serially diluted to produce six standard solutions at concentrations of 0.1, 0.4, 0.8, 1.6, 3.2, and 4.0 µg/mL. The mean peak areas obtained from HPLC analysis were plotted against these concentrations to construct a calibration curve, which was assessed using a linear regression model and the corresponding squared correlation coefficient (*R*^2^). A zero-intercept calibration model was used (Figure 2c). An unconstrained fit yielded an intercept of 4.20 (95% CI: −0.02 to 8.43), which did not differ significantly from zero (*p* = 0.051), supporting the use of the zero-intercept model over the studied range. Calibration standards were prepared from HPLC-grade buserelin (Sigma-Aldrich Fine Chemicals Biosciences, St. Louis, MO, USA), which is supplied with a certificate of analysis confirming compound purity. The calibration curve demonstrated excellent linearity across six concentration levels (0.1–4.0 µg/mL), with a coefficient of determination (*R*^2^) of 0.99 for the zero-intercept model. Residuals from the fitted line were small relative to the response and showed no systematic trend across the concentration range.

Calibration curves were generated by plotting HPLC peak areas against the corresponding buserelin concentrations. The steady-state flux (*J*_ss_), representing the constant rate of drug permeation through the membrane, was determined from the linear portion of the cumulative permeation–time curve using the equation *J*_ss_ = d*Q*/(*A* × d*t*), where *J*_ss_ is the steady-state flux (µg/cm^2^·h), d*Q*/d*t* is the slope of the linear portion of the permeation curve (µg/h), and *A* is the effective diffusion area of the tissue (cm^2^). The apparent permeability coefficient (*P*_app_) was calculated from the steady-state flux and the initial donor concentration (*C*_0_) using the equation *P*_app_ = *J*_ss_/*C*_0_, where *P*_app_ is expressed in cm/h, *J*_ss_ in µg/cm^2^·h, and *C*_0_ in µg/mL. The percentage permeability (*x*) was determined using the equation *x* = ((*T*_s_ × *V*)/*OC*) × 100, where *x* is the cumulative proportion of drug permeated through the endometrial tissue (%), *T*_s_ is the concentration of buserelin in the receptor sample (µg/mL), *V* is the total receptor volume in the Franz cell (mL), and *OC* is the initial amount of buserelin in the donor compartment (µg). In addition to steady-state flux, the instantaneous flux (*J*_t_) was calculated for each sampling interval to compare diffusion behavior between treatment groups.

### 2.5. Statistical Analysis

Statistical analyses were performed using SAS software version 9.4 (SAS Institute Inc., Cary, NC, USA). Descriptive statistics, including means and standard deviations, were generated using the MEANS procedure. Differences in buserelin concentration across sampling times and between treatment groups were analyzed using a general linear mixed model (MIXED procedure). The model included the fixed effects of treatment group, sampling time, and their interaction on the dependent variable (buserelin concentration). Sample identity was included as a random effect to account for repeated measurements within each tissue sample. Both mixed-effects models converged successfully and yielded positive sample-level and residual variance estimates, supporting numerical stability. The estimated intraclass correlations were 0.32 for concentration and 0.23 for flux. A sensitivity analysis omitting the random effect yielded the same conclusions. Because tissue portions from the same sow were allocated to both treatments, the experiment had an inherent paired structure. Overall percentage permeation at 8 h, the proportion of buserelin retained within the tissue, residual drug in the donor chamber, and system loss were compared between treatments using a paired Student’s *t*-test (TTEST procedure), in which the two tissue portions obtained from the same sow served as matched pairs. Normality of the paired differences was assessed using the Shapiro–Wilk test, and Wilcoxon signed-rank tests were performed as a non-parametric sensitivity analysis. In the mixed-model analysis, this pairing was accounted for by including sample identity as a random effect. Least-squares means were calculated for each main effect and interaction. Comparisons between groups and sampling times were performed using the Tukey–Kramer adjustment for multiple comparisons. Statistical significance was defined as *p* < 0.05.

## 3. Results

The linearity of the calibration curve for standard buserelin was confirmed across all tested concentrations, demonstrating excellent analytical precision (*R*^2^ = 0.99) when the regression intercept was set to zero (Figure 2c). The mean pH values of the buserelin solution and the buserelin solution supplemented with diluted semen were 6.14 and 6.81, respectively. The overall percentage of buserelin permeation through porcine endometrial tissue was similar between the buserelin group (61.3 ± 7.8%; range, 54.0–73.7%) and the buserelin + diluted semen group (62.2 ± 4.6%; range, 48.6–68.9%; *p* = 0.840). Cumulative permeation values for both groups over the 8-h period are summarized in Table 1.

**Figure 3:**
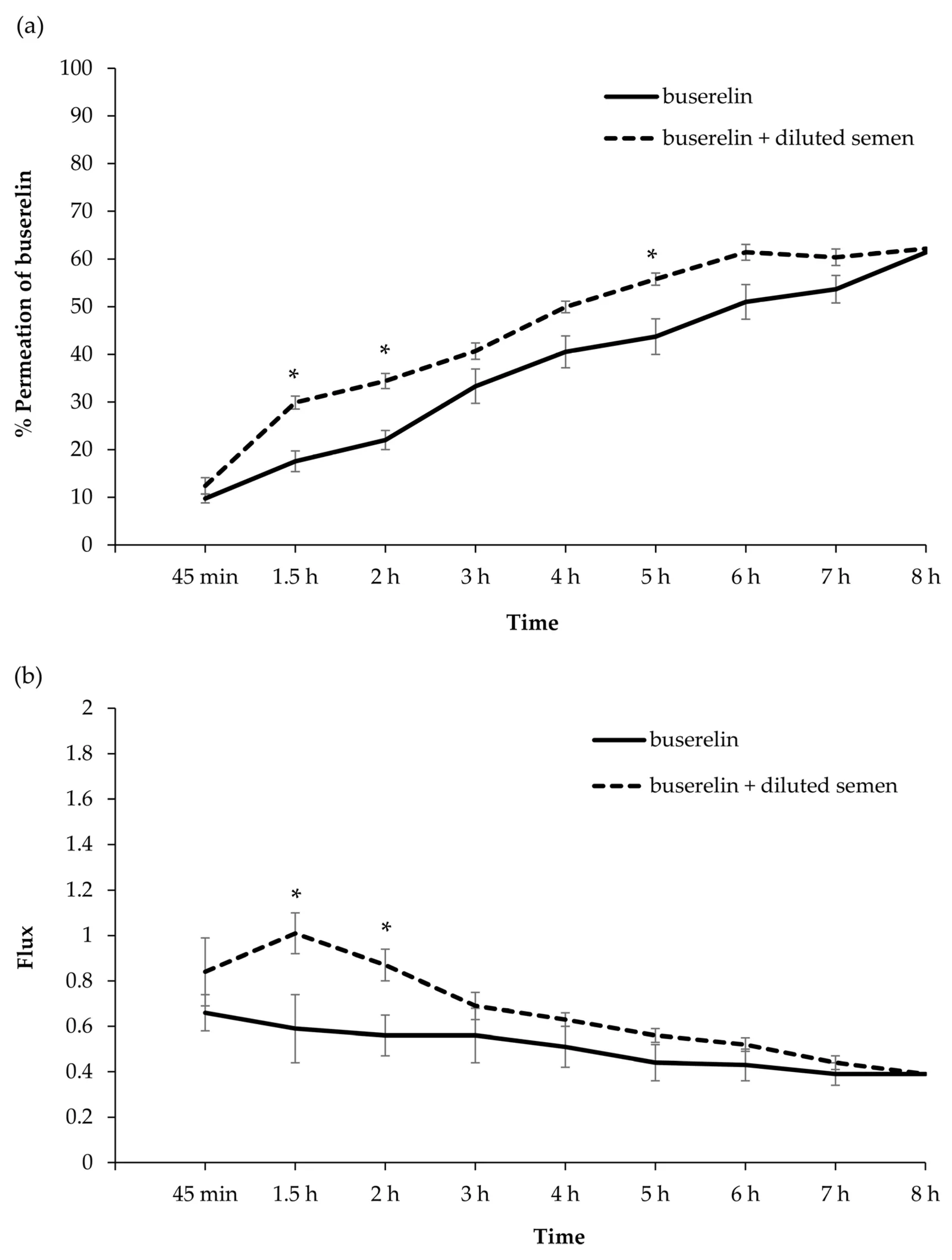
(**a**) Percent permeation (%) and (**b**) instantaneous flux (*J*_t_) of buserelin (µg/cm^2^·h) in the buserelin group and the buserelin plus diluted semen group (least-squares means ± SEM). * Indicates a statistically significant difference between groups (*p* < 0.05).

Cumulative permeation percentages were similar between the buserelin and buserelin + diluted semen groups during the first 0.75 h (0–45 min; 10.2 ± 2.4% vs. 11.8 ± 2.4%, *p* = 1.000). At 1.5 h, however, cumulative permeation was significantly higher in the buserelin + diluted semen group than in the buserelin group (29.2 ± 2.4% vs. 18.0 ± 2.4%, *p* = 0.032). Higher cumulative permeation was also observed in the buserelin + diluted semen group at 2 and 5 h, whereas no significant differences were detected between groups at 3, 4, 6, 7, or 8 h (Figure 3a).

The steady-state fluxes of buserelin across the endometrial tissue were estimated to be 0.21 µg/cm^2^·h for the buserelin group (*R*^2^ = 0.98) and 0.28 µg/cm^2^·h for the buserelin + diluted semen group (*R*^2^ = 0.99). Instantaneous flux values compared at each time point showed that during the first 2 h, the flux of buserelin diluted with semen was higher than that of buserelin alone (*p* < 0.05; Figure 3b). Thereafter, no significant differences were observed between groups from 3 to 8 h (*p* > 0.05). The apparent permeability coefficients were 0.052 cm/h for the buserelin group and 0.069 cm/h for the buserelin + diluted semen group. By the 8-h time point, the proportion of buserelin accumulated within the endometrial tissue remained statistically similar between the buserelin and buserelin + diluted semen groups (17.9 ± 3.2% vs. 16.0 ± 1.7%; *p* = 0.210). The residual drug remaining in the donor chamber accounted for 19.5% in the buserelin group and 18.6% in the buserelin + diluted semen group (*p* = 0.660), whereas overall drug loss from the system was minimal, at approximately 2.6% and 3.2%, respectively (*p* = 0.729).

## 4. Discussion

Overall, findings from this ex vivo pilot model showed that more than 61% of buserelin permeated porcine endometrial tissue within 8 h, regardless of semen supplementation. The penetration of buserelin combined with diluted semen varied among sows, ranging from 48.6% to 68.9%, indicating that a measurable amount of buserelin was able to permeate the endometrium in all animals. On average, the interval from intramuscular administration of buserelin to ovulation is approximately 37.5 ± 3.2 h [24]. Thus, achieving a 61% penetration of buserelin within 8 h after artificial insemination may be compatible with the expected timing of ovulation. Moreover, up to ≈12% of buserelin mixed with boar semen permeated through the endometrial tissue within 45 min after insemination, representing an early fraction whose sufficiency to initiate the ovulatory process is unknown in sows. The faster permeation observed during the early hours with the semen-containing formulation may indicate potential accumulation within reproductive tissues [6,7], although no pharmacokinetic or pharmacodynamic measurements were performed in this study. However, because this experiment was performed ex vivo using tissues from a limited number of sows, the findings should be interpreted as evidence of tissue permeation rather than proof of in vivo efficacy, ovulation induction, or reproductive benefits. Therefore, the early increase observed with diluted semen should be regarded as a hypothesis-generating, formulation-related finding that warrants further investigation.

Buserelin permeation across porcine endometrial tissue increased progressively, reaching 2.46 µg with buserelin alone and 2.49 µg with buserelin plus diluted semen after 8 h. These values were comparable to that previously reported for porcine vaginal tissue using a similar Franz diffusion setup (2.43 µg/cm^2^ at 8 h) [22], indicating effective permeation across both uterine and vaginal mucosa. However, early-phase diffusion (≤3 h) was slower in the present study (0.39–0.50 µg at 1 h) than that reported by Uddin et al. [22] (1.32 µg/cm^2^), likely because of structural and morphological differences between the endometrial and vaginal epithelia that affect diffusional resistance [16,30]. In modern hyperprolific sows, the uterus is approximately 348.3 cm long, whereas the vagina is approximately 13.0 cm long [31]. Therefore, administering buserelin directly into the uterine horns may enhance absorption because their larger surface area may allow for more extensive tissue penetration than intravaginal administration.

The higher flux observed during the first 2 h in the buserelin plus diluted semen group indicates that diluted semen facilitated early drug diffusion, possibly by affecting tissue hydration or interfacial interactions. However, these mechanisms were not evaluated in the present study and require further investigation. Tissue hydration is known to increase molecular mobility and reduce mucosal resistance to peptide diffusion [32,33,34]. After approximately 3 h, both groups reached steady-state conditions, at which point the influence of formulation diminished and transport was governed primarily by the physicochemical properties of buserelin and the intrinsic barrier characteristics of the endometrium [35,36]. In contrast, the lower initial permeability observed in the buserelin-alone group may be attributed to interfacial adsorption, as positively charged peptide molecules can adhere to negatively charged glycoproteins and proteoglycans within the endometrial matrix [37,38]. Specific binding of buserelin to GnRH receptors on the endometrial membrane could further reduce the concentration of free peptide available for diffusion [37,39]. When buserelin is mixed with diluted semen, proteins and GnRH-like peptides may compete for binding sites or stabilize the peptide in solution, thereby reducing nonspecific adsorption and producing a more consistent diffusion profile [39,40,41]. Furthermore, the slightly higher pH of the semen-supplemented formulation (6.81 vs. 6.14) may have favored peptide permeation. The near-neutral pH observed in the formulation containing diluted boar semen may reduce the protonation of ionizable residues, such as guanidino and imidazole groups, potentially increasing membrane affinity and facilitating permeation [41,42,43,44,45,46]. However, this possibility was not directly tested because pH was measured as a characteristic of each formulation rather than experimentally controlled. Therefore, the potential effect of pH on permeation remains hypothetical. Moreover, the two formulations differed in buserelin concentration, donor volume, pH, and semen content. Thus, the observed differences should be interpreted as formulation-level effects rather than effects specifically attributable to semen.

The percentage permeated was calculated by dividing the cumulative amount permeated at 8 h by the initial donor dose. In the present study, 2.46 µg permeated from an applied dose of 4 µg (approximately 61%), whereas Uddin et al. [22] reported 1.83 µg/cm^2^ at 8 h from a donor solution containing 40 µg/cm^3^ of buserelin, corresponding to approximately 5% of the applied dose. This comparison suggests diffusion-limited transport after tissue saturation is reached [36,47]. Collectively, these findings indicate that semen or its extender may enhance permeation during the early diffusion phase, whereas subsequent transport is governed primarily by the physicochemical properties of buserelin and the structural characteristics of the endometrial barrier.

Although semen quality was not assessed in the present study, our previous work showed that supplementation with 5 µg of buserelin did not adversely affect total or progressive motility, sperm viability, acrosome integrity, or plasma membrane integrity at 0 and 6 h after supplementation [8]. That study used BTS rather than MR-A^®^ 3, the extender used here. Both extenders are designed to support sperm viability and motility during semen storage; however, whether the extender influences sperm responses to buserelin remains to be investigated. In the present study, buserelin crossed the endometrial tissue both alone and when mixed with extended semen. This finding supports the feasibility of diffusion in the presence of semen, but the contribution of semen quality to this process cannot be determined from the current experimental design.

This study quantified the proportions of buserelin that permeated and were retained within the tissue or remained in the donor chamber, providing evidence that buserelin can cross the porcine uterine lining. This permeation supports the biological plausibility of intrauterine buserelin delivery, although the amount required to induce an LH surge and trigger ovulation in vivo remains to be determined. The improved litter size reported after adding buserelin to diluted semen under field conditions [6,8] further supports its potential reproductive benefits; however, additional studies are needed to link the permeated dose with the physiological threshold for ovulation induction.

An important strength of the experimental design was that both treatments contained the same total buserelin dose (4 µg) while representing different application conditions. The buserelin plus diluted semen group had a larger donor volume than the buserelin-only group (2 vs. 1 mL), resulting in a lower initial buserelin concentration within a physiologically relevant semen matrix. This design reflects standard on-farm practice, in which hormones are added directly to extended semen. Therefore, the early differences in permeation likely reflect the combined effects of donor concentration and the semen matrix. These findings should be interpreted in the context of realistic application conditions and provide useful insight into buserelin transport when administered with extended semen.

Although this ex vivo study provides new insights into buserelin permeation across porcine endometrial tissue, some limitations should be acknowledged. The Franz diffusion cell system does not fully replicate the dynamic physiological environment of the reproductive tract, including blood perfusion, enzymatic activity, and hormonal fluctuations during estrus, all of which may influence in vivo absorption. In addition, the number of tissue samples was limited. Nevertheless, we evaluated the precision and minimum detectable effect size achieved with the available sample size. The 95% confidence interval for the between-formulation difference had a constant width of 0.86 µg, and the smallest difference detectable with 80% power was estimated to be 0.62 µg. Thus, the study was able to detect relatively large differences despite its limited sample size; however, the time-specific comparisons should be interpreted as exploratory. Moreover, only endometrial tissue was examined; other potential absorption sites, such as the cervix and oviduct, were not assessed. Although the composition and pH of the diluted semen were controlled, the biochemical properties of semen may vary under field conditions. Thus, these findings provide mechanistic evidence that buserelin can permeate porcine endometrial tissue but should not be interpreted as evidence of in vivo efficacy or reproductive benefits. Further in vivo studies evaluating follicular dynamics by ultrasonography, characterizing the LH surge, and determining the minimum effective intrauterine GnRH dose are needed before these findings can be translated into practical reproductive recommendations. However, evaluating the effectiveness of intrauterine GnRH in inducing an LH surge in vivo would require frequent blood sampling to capture the pulsatile nature of LH secretion. External jugular vein catheterization could facilitate serial blood collection in gilts [48].

Frozen tissue can be used in ex vivo Franz diffusion studies, as supported by previous research [11,22,27,28]. Studies have shown that storage at −20 °C can preserve tissue structure and yield reliable permeation data [11,22]. Amores et al. [27] reported no significant differences in flux, permeability coefficient, or lag time between fresh porcine buccal mucosa and tissue cryopreserved at −80 °C for up to one month under buffered conditions. Iliopoulos et al. [28] also documented the use of frozen tissue in Franz diffusion experiments. Together, these findings support frozen tissue as a practical option for permeation studies, although its suitability depends on the tissue type and storage protocol. In the present study, endometrial tissues were stored in buffer at −20 °C and used within 48–72 h to limit deterioration. For passive permeation studies, preservation of tissue barrier integrity is more directly relevant than cellular viability alone. Both formulations were evaluated using sections from the same tissue maintained under identical storage conditions, helping to control storage-related variability. Although these precautions do not exclude potential effects of freezing on absolute permeation values, the available evidence and standardized handling support the use of frozen endometrial tissue as a relevant experimental model for the preliminary assessment of buserelin passage across the uterine lining.

## 5. Conclusions

This ex vivo pilot study demonstrated efficient buserelin permeation across porcine endometrial tissue, with more than 61% of the administered dose permeating within 8 h. Semen supplementation enhanced early-phase permeation, while overall permeation was comparable by 8 h. These preliminary findings support further in vivo investigation of buserelin delivery with extended semen.

## Figures and Tables

**Table 1:** Cumulative buserelin permeation values for the buserelin and buserelin plus diluted semen groups over an 8-h diffusion period using Franz diffusion cells (mean ± SD, µg).

Time Point	Buserelin	Buserelin + Diluted Semen	*p* Value
45 min	0.39 ± 0.16	0.50 ± 0.15	1.000
1.5 h	0.70 ± 0.09	1.19 ± 0.17	0.032
2 h	0.88 ± 0.21	1.38 ± 0.13	0.029
3 h	1.33 ± 0.20	1.63 ± 0.16	0.810
4 h	1.62 ± 0.35	2.00 ± 0.17	0.340
5 h	1.75 ± 0.33	2.23 ± 0.12	0.040
6 h	2.04 ± 0.36	2.46 ± 0.13	0.174
7 h	2.15 ± 0.36	2.41 ± 0.16	0.912
8 h	2.46 ± 0.28	2.49 ± 0.17	1.000

## Data Availability

The data presented in this study are available on request from the corresponding author.
